# Intergenic Transcription, Cell-Cycle and the Developmentally Regulated Epigenetic Profile of the Human Beta-Globin Locus

**DOI:** 10.1371/journal.pone.0000630

**Published:** 2007-07-18

**Authors:** Joanne Miles, Jennifer A. Mitchell, Lyubomira Chakalova, Beatriz Goyenechea, Cameron S. Osborne, Laura O'Neill, Keiji Tanimoto, James Douglas Engel, Peter Fraser

**Affiliations:** 1 Laboratory of Chromatin and Gene Expression, The Babraham Institute, Babraham Research Campus, Cambridge, United Kingdom; 2 Institute of Biomedical Research, The Medical School, University of Birmingham, Birmingham, United Kingdom; 3 Department of Cell and Developmental Biology, University of Michigan Medical School, Ann Arbor, Michigan, United States of America; Institut Curie, France

## Abstract

Several lines of evidence have established strong links between transcriptional activity and specific post-translation modifications of histones. Here we show using RNA FISH that in erythroid cells, intergenic transcription in the human *β*-globin locus occurs over a region of greater than 250 kb including several genes in the nearby olfactory receptor gene cluster. This entire region is transcribed during S phase of the cell cycle. However, within this region there are ∼20 kb sub-domains of high intergenic transcription that occurs outside of S phase. These sub-domains are developmentally regulated and enriched with high levels of active modifications primarily to histone H3. The sub-domains correspond to the *β*-globin locus control region, which is active at all developmental stages in erythroid cells, and the region flanking the developmentally regulated, active globin genes. These results correlate high levels of non-S phase intergenic transcription with domain-wide active histone modifications to histone H3.

## Introduction

Far more transcription takes place in the cell nucleus than can be accounted for by protein-coding gene transcription alone[1]–[5]. Transcriptome studies have revealed a plethora of non-coding RNAs, some of which have been implicated in diverse regulatory processes such as dosage compensation, genomic imprinting and RNAi. However, most non-genic transcripts seem to fall into a category characterized by several studies investigating intergenic transcription[6]. Intergenic transcripts are often produced from regions flanking active genes and their long-range regulatory elements. Their occurrence in correlation with gene activity and chromatin structural alterations, suggests a role in the regulation of gene expression. Such intergenic transcription has been most extensively studied in the *Drosophila* bithorax complex and human *β*-globin loci. In the bithorax complex it has been suggested that intergenic transcription plays a role in initiating activation of the locus[7] and in regulating cellular memory[8]. Intergenic transcription has also been proposed to be involved in regulation of the major histocompatibility complex locus[9], the human growth hormone locus[10] the IL4 locus[11], the IL10 gene cluster[12], regulating chromatin accessibility during VDJ recombination[13], and in regulating probability of choice of X chromosome inactivation[14]. Thus intergenic transcription is common and may be a part of varied mechanisms for the regulation of gene expression in eukaryotes.

In the human *β*-globin locus intergenic transcripts can be found throughout the locus in erythroid cells[15], [16] at very low abundance relative to gene transcripts. RNA FISH (fluorescent *in situ* hybridisation) showed that intergenic transcripts are only detected in a proportion of cells in an unsynchronized population. They appear to be generated in a cell-cycle-dependent manner; detectable predominantly during G1 phase with a small percentage of loci showing RNA FISH signals in early S-phase[16]. The highest percentage of loci with positive signals are detected with probes homologous to the DNase I sensitive sub-domains containing the locus control region (LCR) and active adult *β*-globin genes (*HBD*, haemoglobin delta; *HBB*, hemoglobin beta). Deletion of a 2.5 kb region containing the putative adult sub-domain intergenic promoter results in a sub-domain-wide failure to adopt the characteristic DNase I sensitive chromatin conformation during development, and an abnormally low and variegated expression of the adult *HBB* gene[16], [17]. These results suggest that intergenic transcription could play a role in decondensation of chromatin domains and gene activation.

Genome scale studies have revealed that histone H3 di- and tri-methylation on lysine 4 (H3K4me2, H3K4me3) as well as H3 and H4 hyperacetylation (H3ac and H4ac, respectively) are enriched in the regions surrounding active genes[18]–[20]. Tri-methylation on H3K4 correlates most strongly with the promoter regions of expressed genes, often residing within 1 kb of the transcription start site. Hyperacetylation of both H3 and H4 also correlate with promoter regions while di-methylation on H3K4 occurs across wider regions in the vicinity of active genes. In contrast to the punctate patterns of H3K4 methylation detected in most active regions, several of the *Hox* clusters contain large domains of enriched H3K4 methylation encompassing multiple genes and their flanking sequences. *Hox* H3K4 methylated domains appear to be tissue-specific and correlate significantly with intergenic transcription[19].

A complex pattern of histone modifications has been detected in the human and mouse *β*-globin loci[21]–[26]. Collectively, these experiments have suggested a correlation between H3 and H4 acetylation and H3K4 methylation modifications associated with active genes, and DNase I hypersensitivity sites in the LCR as well as areas of high general DNase I sensitivity. Here we present a high-resolution, locus-wide analysis of intergenic transcripts and histone modifications across the human *β*-globin locus during development in yeast artificial chromosome (YAC) transgenic mice and in primary human erythroid cells. We show that the intergenic transcription pattern is complex and extensive and that active histone modifications strongly correlate with areas of non-S phase intergenic transcription linking the cell-cycle-specific timing of intergenic transcription with large chromatin domains of modified histones.

## Results

### Intergenic transcription throughout the human *β*-globin locus

We previously identified regions of intergenic transcription in the human *β*-globin locus in transgenic mice via RNA FISH[16]. Though rather crudely mapped, regions of increased intergenic transcription corresponded to domains of increased general DNase I sensitivity. To verify our FISH data and obtain higher resolution we used quantitative real-time reverse transcription PCR (RT-PCR) with several primer pairs encompassing nearly all of the non-repetitive, non-genic sequences across the entire *β*-globin locus. Total RNA was prepared for analysis from embryonic day E11.5 red cells and adult anemic spleen from transgenic 264W which contains a single copy human *β*-globin locus YAC[27].

**Figure 1:**
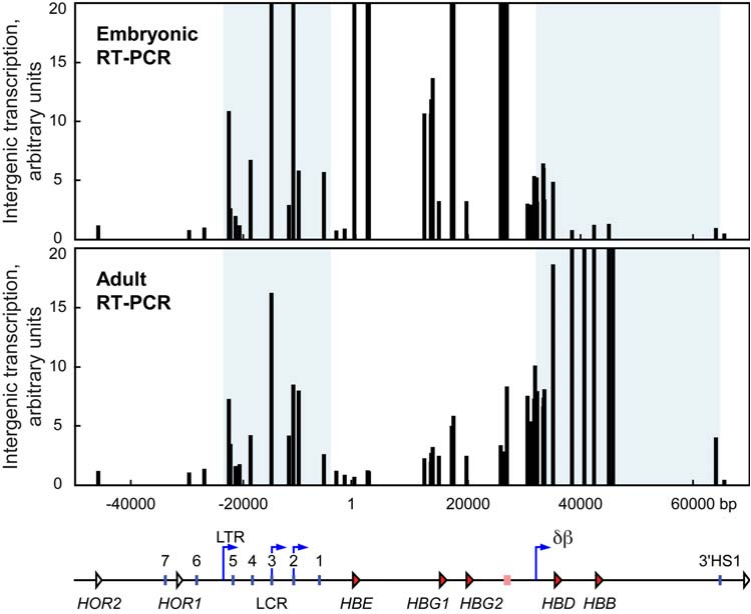
Distinct domains of intergenic transcription in the human *β*-globin locus are developmentally regulated. Intergenic transcription in the human *β*-globin locus in 264W transgenic mice measured by quantitative RT-PCR. Total RNA was prepared from erythroid tissues at two developmental stages – E11.5 embryonic blood (Embryonic) and adult anemic spleen (Adult). cDNA was produced and quantified by real-time PCR using primer pairs across the human *β*-globin locus. Bar plots represent relative transcript quantities normalised to the most 5′ primer pair in the olfactory receptor gene region, which shows low levels of transcription at both developmental stages. Primer pair positions are relative to the *HBE* gene transcription start site at position 1; they are aligned with a map of the locus shown below the graphs. Shaded regions of the graphs correspond to the locus control region (LTR promoter to downstream of LCR HS1) and the δβ domain (δβ intergenic promoter to 3′HS1). Map features: red arrowheads, globin genes; pink rectangle, *β*-like pseudogene; white arrowheads, olfactory receptor genes (*HOR*, human olfactory receptor); vertical blue lines, hypersensitive sites; blue arrows, intergenic transcription start sites; LTR, long terminal repeat; δβ, δβ intergenic promoter.

The results show that at both stages of development the LCR is transcribed (Figure 1), consistent with our previous RNA FISH findings in which a relatively large percentage of loci were positive for transcript signals in this region. The abundance of LCR transcripts appears to be greater in embryonic blood cells compared to adult anemic spleen cells, which may reflect the fact that embryonic blood is composed of nearly 100% erythroid cells, whereas adult anemic spleen is 80–90% erythroid. However, we cannot rule out the possibility, and in fact the data suggest, that the intergenic transcription pattern in the LCR region may change during development. This is consistent with the LCR's role as a complex regulatory region[28], [29] with many intergenic promoters[30]–[33]. In embryonic cells intergenic transcript levels are high throughout the majority of the εγ domain whilst relatively low in the δβ domain. The region of low transcript levels immediately upstream of the *ΗΒΕ* (hemoglobin epsilon) gene suggests that the majority of LCR transcripts are not contiguous with εγ domain transcripts.

In adult cells transcript levels in the εγ domain are reduced whilst the LCR and δβ domains are relatively highly transcribed. The exceptions to this pattern are the regions immediately upstream and downstream of the δβ promoter where similar levels are seen at both stages of development. In addition to sense transcription in this region, strand-specific RT-PCR detects significant levels of antisense transcription, which appears to initiate somewhere downstream of the δβ promoter and terminate somewhere in the vicinity of the *β*-like pseudogene (unpublished observations). These data showing high levels of intergenic transcription in the LCR and the δβ domain in adult cells are consistent with our RNA FISH data in which a larger percentage of cells displayed RNA FISH signals in these regions, compared to the εγ domain[16].

### Developmentally regulated histone modifications in the *β*-globin YAC transgene locus

**Figure 2:**
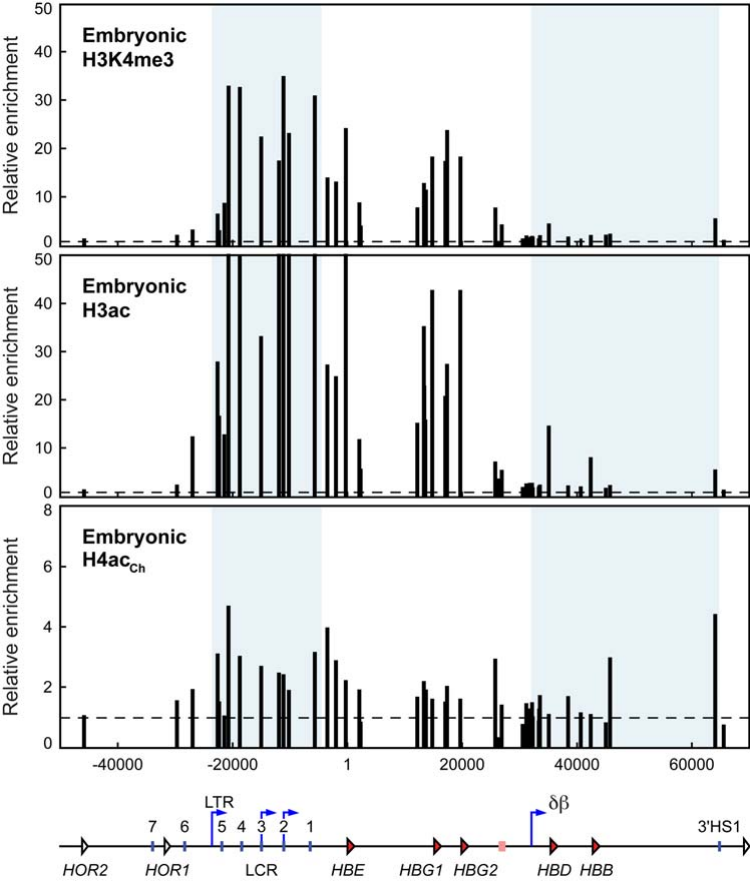
Histone modifications throughout the human *β*-globin locus in embryonic cells reflect intergenic transcription patterns. Histone modifications in the human *β*-globin locus in embryonic blood cells from 264W transgenic embryos assayed by ChIP. Chromatin from E11.5 embryonic blood cells was immunoprecipitated with antibodies specific for trimethylated lysine 4 of histone H3 (H3K4me3), acetylated histone H3 (K9/14, H3ac), and acetylated histone H4 (ChIP grade antibody, K5/18/12/16, H4ac_Ch_). The fold-enrichment of antibody-bound sequences over input was analysed by real-time PCR using primer pairs across the *β*-globin locus. Bar plots represent enrichment normalised to the most 5′ primer pair in the olfactory receptor gene region, which shows low enrichment for all antibodies; horizontal dashed lines mark the level of the normalisation data point (value 1). Primer pair positions are relative to the *HBE* gene transcription start site at position 1; they are aligned with a map of the locus shown below the graphs. Shaded regions of the graphs correspond to the locus control region (LTR promoter to downstream of LCR HS1) and the δβ domain (δβ intergenic promoter to 3′HS1). Map features: red arrowheads, globin genes; pink rectangle, *β*-like pseudogene; white arrowheads, olfactory receptor genes (*HOR*, human olfactory receptor); vertical blue lines, hypersensitive sites; blue arrows, intergenic transcription start sites; LTR, long terminal repeat; δβ, δβ intergenic promoter.

To compare histone modifications within the human *β*-globin locus to levels of intergenic transcription we assessed the pattern of histone modifications across the human *β*-globin locus in the 264W line using native chromatin immunoprecipitation (ChIP) on erythroid cells from E11.5 embryonic blood and adult anemic spleen. We sought to generate a high-resolution map of active histone modifications in the human *β*-globin locus in transgenic mice. In E11.5 embryonic blood, the LCR and the εγ domain are highly enriched for histone H3K4 tri-methylation and histone H3 hyperacetylation (Figure 2, top and middle panel). Conversely, the δβ domain is relatively devoid of these histone modifications. The 5′ and 3′ boundaries of enrichment for these active modifications occur near the long terminal repeat (LTR) promoter upstream of hypersensitive site 5 of the LCR (HS5) and the region upstream of the δβ intergenic promoter. This H3K4me3 and H3ac domain correlates well with the domain of increased intergenic transcription at E11.5 (Figure 1, top panel). In contrast to H3K4me3 and H3 hyperacetylation, H4 acetylation appears to be moderately enriched across the entire locus and does not appear to follow the same domain structure (Figure 2, bottom panel).

**Figure 3:**
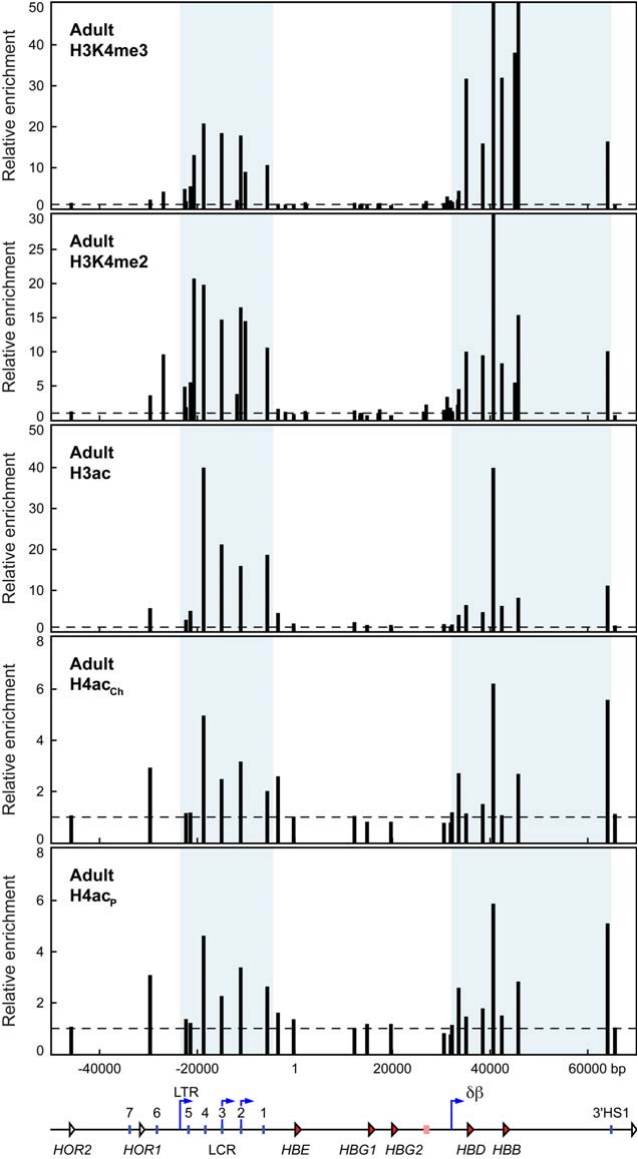
Histone modifications throughout the human *β*-globin locus in adult cells reflect intergenic transcription patterns. Histone modifications in the human *β*-globin locus in 264W transgenic adult erythroid cells assayed by ChIP. Chromatin from anemic spleen was immunoprecipitated with antibodies specific for trimethylated lysine 4 of histone H3 (H3K4me3), dimethylated lysine 4 of histone H3 (H3K4me2), acetylated histone H3 (K9/14, H3ac), and acetylated histone H4 (two different antibodies: ChIP grade antibody, K5/18/12/16, H4ac_Ch_; penta lysine, H4ac_P_). The fold-enrichment of antibody-bound sequences over input was analysed by real-time PCR using primer pairs across the *β*-globin locus. Bar plots represent enrichment normalised to the most 5′ primer pair in the olfactory receptor gene region, which shows low enrichment for all antibodies; horizontal dashed lines mark the level of the normalisation data point (value 1). Primer pair positions are relative to the *HBE* gene transcription start site at position 1; they are aligned with a map of the locus shown below the graphs. Shaded regions of the graphs correspond to the locus control region (LTR promoter to downstream of LCR HS1) and the δβ domain (δβ intergenic promoter to 3′HS1). Map features: red arrowheads, globin genes; pink rectangle, *β*-like pseudogene; white arrowheads, olfactory receptor genes (*HOR*, human olfactory receptor); vertical blue lines, hypersensitive sites; blue arrows, intergenic transcription start sites; LTR, long terminal repeat; δβ, δβ intergenic promoter.

In adult anemic spleen we took advantage of the hugely increased numbers of available erythroid cells and used additional antibodies against di-methylated H3K4 and penta-acetylated H4 (H4ac_p_) in addition to those above. The results show that the histone modification profile in adult erythroid cells is dramatically different compared to E11.5 cells. The LCR and the δβ domain are highly enriched for H3K4me3, H3K4me2 and H3ac (Figure 3), while the εγ domain lacks these histone modifications. The presence of these histone modifications in the LCR and δβ domains correlates with increased intergenic transcription in these regions in adult cells (Figure 1, bottom panel) and increased general sensitivity to DNase I [16]. A sub-domain pattern of H4 acetylation is discernable with both H4 antibodies (Figure 3) but appears to be less well defined compared to H3 modifications, as in embryonic cells. The LCR and δβ sub-domains do appear enriched for H4 acetylated chromatin over the inactive εγ domain and the upstream olfactory receptor gene (*ORG*) region.

### Histone modification profile of the endogenous *β*-globin locus in primary erythroid precursor cells

**Figure 4:**
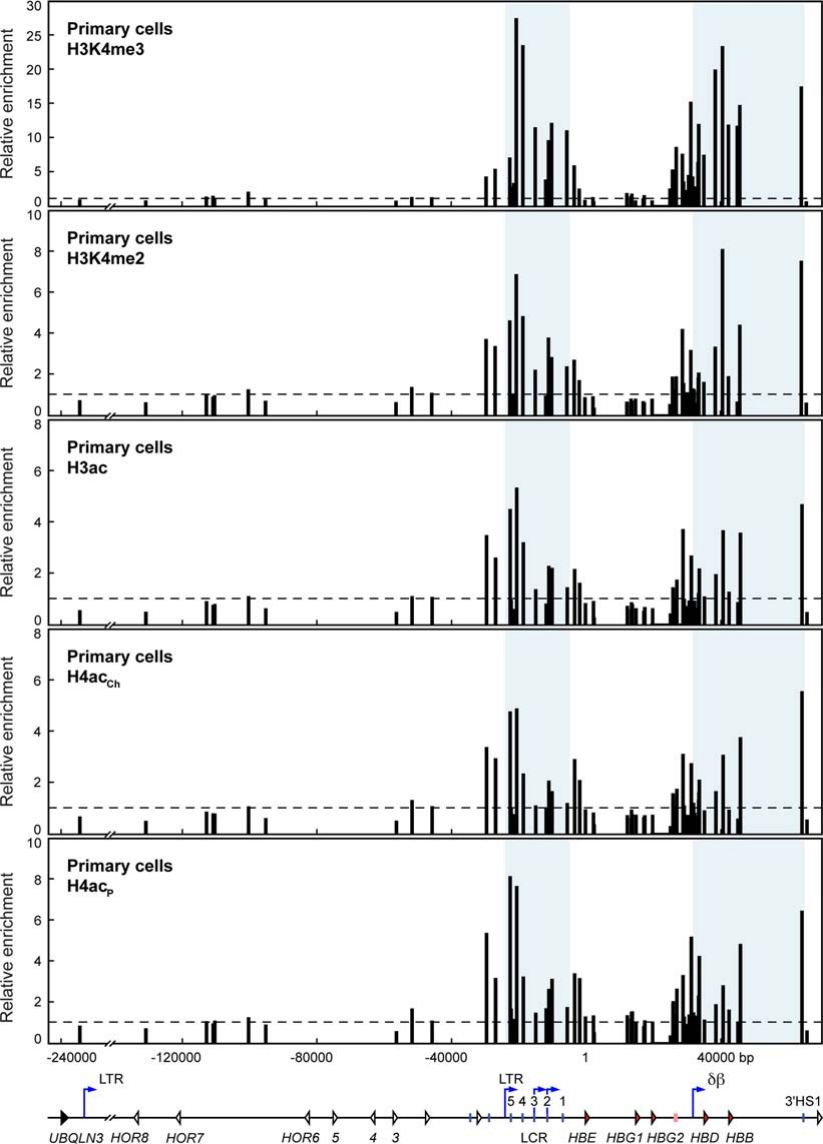
Histone modifications throughout the human *β*-globin locus in primary erythroid precursor cells. Histone modifications in the human *β*-globin locus in human primary erythroid precursor cells assayed by ChIP. Chromatin from erythroid precursor cells was immunoprecipitated with antibodies specific for trimethylated lysine 4 of histone H3 (H3K4me3), dimethylated lysine 4 of histone H3 (H3K4me2), acetylated histone H3 (K9/14, H3ac), and acetylated histone H4 (two different antibodies: ChIP grade antibody, K5/18/12/16, H4ac_Ch_; penta lysine, H4ac_P_). The fold-enrichment of antibody-bound sequences over input was analysed by real-time PCR using primer pairs across the *β*-globin locus. Bar plots represent enrichment normalised to the same primer pair in the olfactory receptor gene region as in previous figures (second from left to right; an additional primer pair upstream of the normalisation point is included in this experiment). Horizontal dashed lines mark the level of the normalisation data point (value 1). Primer pair positions are relative to the *HBE* gene transcription start site at position 1; they are aligned with a map of the locus shown below the graphs. Shaded regions of the graphs correspond to the locus control region (LTR promoter to downstream of LCR HS1) and the δβ domain (δβ intergenic promoter to 3′HS1). Map features: red arrowheads, globin genes; pink rectangle, *β*-like pseudogene; white arrowheads, olfactory receptor genes (*HOR*, human olfactory receptor); vertical blue lines, hypersensitive sites; blue arrows, intergenic transcription start sites; LTR, long terminal repeat; δβ, δβ intergenic promoter.

The human *β*-globin locus has been studied extensively in transgenic mice. Despite the fact that the *β*-globin genes are fully expressed and the locus is developmentally regulated in transgenic mice there have been noticeable differences in the way the human locus is regulated in mice. We were therefore interested in characterising the epigenetic profile in the endogenous human *β*-globin locus at high resolution. Studying the locus in its endogenous chromosomal location provided the additional advantage of extending our analyses further into the 5′*ORG* region. We obtained nucleated, buffy coat cells from human peripheral blood from a local blood bank and used the two-step liquid culture system [34], [35] to generate large quantities of adult erythroid precursor cells. We previously showed by RNA FISH that upon maturation, these cells transcribe primarily *HBB* with a small percentage of loci (approximately 10%) positive for *HBG* (hemoglobin gamma) transcription[6]. We used the same antibodies as in the adult anemic spleen transgenic studies above (Figure 4).

As in the transgenic mice, there are clearly detectable domains of active histone modifications in the LCR and *δβ* domains indicating that in general the adult *β*-globin transgene locus has a domain structure similar to the endogenous *β*-globin locus. However, notable exceptions are the increase in active histone modifications in the region upstream of the *δβ* promoter to the *β*-like pseudogene region. This is apparent for both H3K4me2 and H3K4me3 as well as H3ac and H4ac. This corresponds roughly to the areas of antisense intergenic transcription seen in transgenic mice (unpublished observations). Antisense transcription in this region has also been detected in primary erythroid precursor cells[25]. Also noteworthy is the fact that the εγ domain and region upstream of the *β*-globin locus containing the olfactory receptor gene cluster have low levels of acetylated histones.

### Intergenic transcription through the olfactory receptor gene cluster in erythroid cells

**Figure 5:**
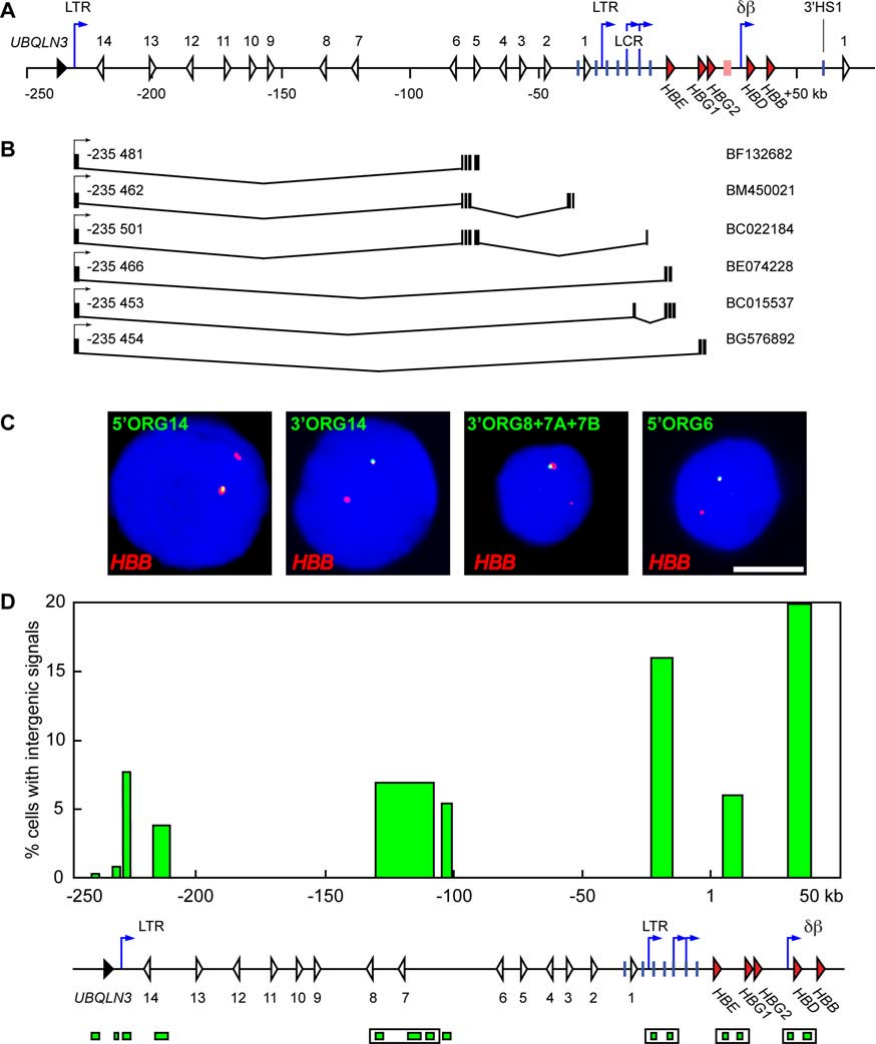
Non-coding transcripts originate 230 kb upstream of the *β*-globin locus. (A) A schematic representation of the human *β*-globin locus. Red arrowheads, globin genes; pink rectangle, *β*-like pseudogene; white arrowheads, olfactory receptor genes; black arrowhead, *UBQLN3* gene; vertical blue lines, hypersensitive sites; blue arrows, intergenic transcription start sites; LTR, long terminal repeats; δβ, δβ intergenic promoter; coordinates in kilobases (kb) are relative to the *HBE* gene transcription start site at position 1. (B) ESTs originating from the LTR promoter 235 kb upstream of the *ε* gene. Intervening spliced regions are depicted along with the location of exons. Coordinates (in base pairs) of the start sites (arrows) are indicated; NCBI Nucleotide database accession numbers are shown on the right. (C) RNA FISH analysis of intergenic transcription in the 5′ olfactory receptor gene cluster region in human primary erythroid precursor cells. Intergenic transcription signals are detected in green, *HBB* signals are detected in red, DAPI staining of nuclear DNA is in blue. Examples for 3 individual intergenic probes and one probe cocktail are shown, as indicated; for probe labels and positions see (D) and Materials and Methods; scale bar, 5 µm. Each intergenic probe detects signals only in a proportion of all *β*-globin loci in the cell population. The percentage of cells with intergenic signals was scored for individual probes or cocktail; results from the quantitative analysis are presented in (D). (D) Bar plot representing percentages of cells with intergenic signals. Bar positions on the x axis correspond to probe positions and are aligned with the map below the graph; coordinates in kilobases (kb) are relative to the *HBE* gene transcription start site at position 1. Probes are depicted as green boxes below the map. Probe cocktails are indicated by open boxes. Probes from 5′ to 3′: 5′UBQLN3, 3′UBQLN3, 5′ORG14, 3′ORG14, 3′ORG8, 3′ORG7A, 3′ORG7B, 5′ORG6, HS5, HS3, 3′ε, 5′γ, 5′δ, and 5′β. Map features as in (A). Relatively high levels of intergenic transcription are detected in the *β*-globin LCR and the active δβ domain.

Analysis of ESTs and mRNAs in the vicinity of the *β*-globin locus has shown a number of spliced non-coding transcripts with 5′ exons far upstream of the *β*-globin locus and extending into the *β*-globin locus[36]. Many of these transcripts initiate within a 50 bp region within an LTR-like element located just downstream of the *UBQLN3* gene, located approximately 236 kb upstream of the *HBE* gene (Figure 5A and B). We decided to investigate transcription across this region in greater detail using RNA FISH.

We performed double-label RNA FISH in human primary cultured erythroid cells obtained from the two-step liquid culture system described above. We used single-stranded *HBB* intron probes and single-stranded probes to various regions in the *ORG* cluster and *β*-globin locus (Figure 5C). *HBB* intron probes detect gene transcription signals at 90–95% of *β*-globin loci in erythroid cells, and therefore serve as an excellent internal control to identify *HBB* expressing erythroid cells and to mark the position of the *β*-globin locus in the nucleus. RNA FISH with probes immediately 5′ or 3′ of the *UBQULN3* gene, upstream of the -236 LTR, detect little or no transcription signals (Figure 5D). In contrast, probes to the region immediately downstream of the -236 LTR detects significant sense transcripts (7% of loci; p<0.05) (Figure 5D). Similar percentages of positive loci are detected with other probes in the *ORG* region. These results show that the most 5′ transcription start site of sense transcription in the *ORG* cluster is located in the vicinity of the -236 LTR element in primary erythroid cells in agreement with the results of Xiang *et al*.[36] and EST databases. Probes in the LCR and δβ sub-domains detect intergenic transcript signals in three- to five-fold more loci (16–20% versus 4–8%) than the *ORG* probes (Figure 5D), consistent with our previous data in transgenic mice[16] and the RT-PCR data above showing higher levels of RNA transcripts in these regions. Probes in the εγ domain detect signals at approximately 6% of loci in adult erythroid cells, consistent with our earlier observations in transgenic mice. These results show that the percentage of loci in which transcription is occurring in the *ORG* cluster and εγ domains is low while probes in the active domains (LCR and δβ) detect transcription at significantly more loci. Thus, domains of high or frequent intergenic transcription in the human *β*-globin locus correlate strongly with chromatin domains of highly modified chromatin.

### Cell cycle specificity of transcribed domains in the human *β*-globin locus

**Figure 6:**
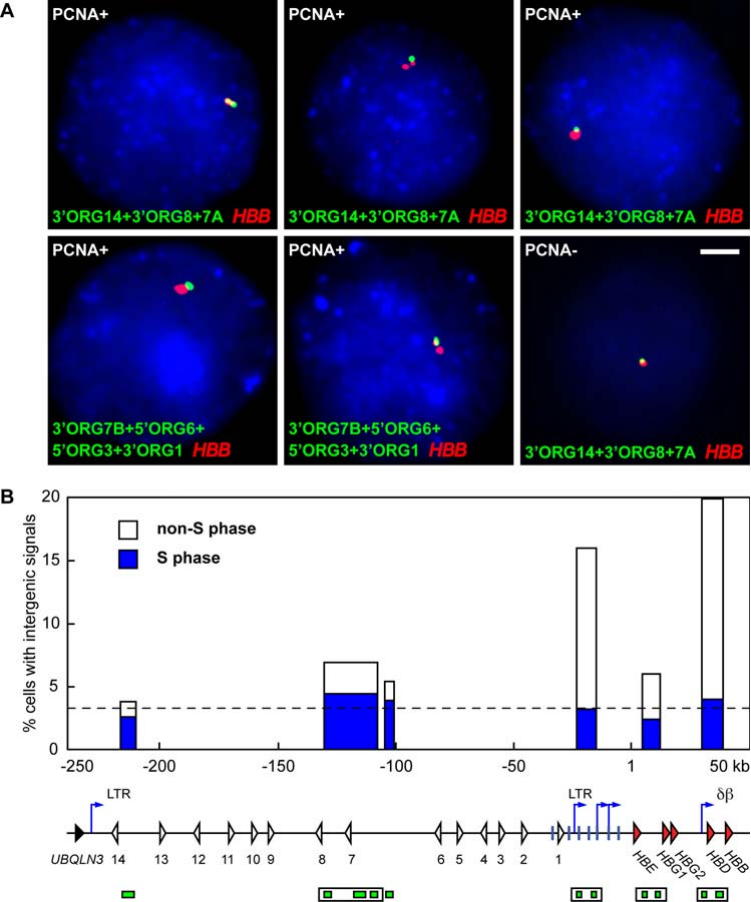
Upstream transcripts occur predominantly in S phase. (A) Cell cycle analysis of intergenic transcription in the 5′ olfactory receptor gene cluster region in human primary erythroid precursor cells by RNA immuno-FISH. Intergenic transcription signals are detected in green, *HBB* transcript signals are red, PCNA is pseudocoloured in blue; scale bar, 2 µm. Shown are single focal planes from several representative PCNA-positive cells and one PCNA-negative cell with various probe combinations. For all probe positions, see Materials and Methods. PCNA-negative cell (bottom right): the brightness of the blue channel is enhanced to demonstrate the absence of PCNA staining. (B) Percentage of PCNA-positive intergenic signal-containing cells was scored for individual probes or the indicated probe cocktails. Bar plot representing percentages of cells with intergenic signals (as in Figure 5D) with the proportion of PCNA-positive cells (S phase) shown in blue. The percentage of intergenic signal-containing S-phase cells is relatively uniform throughout the 5′ *ORG* and *β*-globin loci (average 3.3% marked by dashed line). Bar positions on the x axis correspond to probe positions and are aligned with the map below the graph; coordinates in kilobases (kb) are relative to the *HBE* gene transcription start site at position 1. Probes are depicted as green boxes below the map. Probe cocktails are indicated by open boxes. Probes from 5′ to 3′: 3′ORG14, 3′ORG8, 3′ORG7A, 3′ORG7B, 5′ORG6, HS5, HS3, 3′ε, 5′γ, 5′δ, and 5′β. Map features: red arrowheads, globin genes; pink rectangle, *β*-like pseudogene; white arrowheads, olfactory receptor genes; black arrowhead, *UBQLN3* gene; vertical blue lines, hypersensitive sites; blue arrows, intergenic transcription start sites; LTR, long terminal repeats; δβ, δβ intergenic promoter. The high level of intergenic transcription in the *β*-globin LCR and the active δβ domain is accounted for by higher percentages of non-S-phase cells with intergenic signals.

We previously observed that intergenic transcription is cell-cycle regulated occurring predominantly in G1 phase, but also with a minority of cells showing signals in S phase of the cell cycle[16]. We had previously noted transcription in the *ORG* cluster in erythroid cells derived from human cord blood using RNA FISH probes homologous to a region upstream of the LCR (Gribnau and Fraser, unpublished). However, the transcription signals in this region were unusual. Only a small fraction of cells had signals and many of them appeared as doublets suggesting that the region had been duplicated, indicating that transcription in this area occurs preferentially in S-phase cells[16]. Therefore, we considered the possibility that not only intergenic transcription occurs during restricted stages of the cell cycle, but that different regions are transcribed at different stages. We were interested to determine the cell-cycle timing of transcripts in the upstream *ORG* region compared to the sub-domains of the *β*-globin locus. We used PCNA immuno-staining to mark cells in S phase in conjunction with RNA FISH[16] with intergenic probes in primary cultured human erythroid cells to determine whether these transcripts occur predominantly in S phase or in non-S-phase cells (Figure 6A). The data show that transcription throughout the *ORG* region occurs predominantly in PCNA positive, S-phase cells (Figure 6B). The percentage of signals occurring in S-phase ranges from 64 to 72 in the *ORG* region upstream of the *β*-globin locus. The timing of intergenic transcription in the LCR and δβ sub-domains is markedly different. The majority of signals in these active domains occur in non-S-phase cells. There are still a small percentage of cell nuclei with signals in S-phase nuclei in the active domains and these levels are comparable to the percentage of nuclei with S-phase signals in the *ORG* region. Signals in the εγ sub-domain occur at nearly equal frequencies in S- and non-S-phase nuclei (Figure 6B). These results suggest the possibility that a large transcript that initiates at the -236 LTR element continues through the entire *ORG* region and into the *β*-globin locus in S-phase cells. The existence of these types of transcripts is supported by and consistent with EST data. The increased transcription of the LCR and δβ sub-domains in non-S-phase nuclei shows that the majority of transcription in these regions is controlled independently of transcription in the *ORG* region and εγ sub-domain. Furthermore these results show that the majority of LCR and δβ sub-domain transcripts are not contiguous with each other or the *ORG* and εγ sub-domain transcripts. We noted that the ratio of G1 to S phase intergenic transcription is slightly higher in the εγ domain compared to the *ORG* cluster. We previously showed that a small percentage of cells in the human cell cultures are not fully differentiated, still transcribe the *HBG* genes and would be expected to have an active εγ domain [6]. This may account for the slightly increased ratio of G1/S intergenic transcription in this region.

PCNA localization patterns change as S phase progresses permitting the discrimination of early, middle and late S-phase stages[16], [37]. We noted that with probes to the most 5′ region of the transcribed *ORG* domain we observed a higher proportion of cells in early S phase compared to cells with middle or late PCNA patterns. Probes located more 3′ of the -236 LTR in the *ORG* region detected signals in progressively later S-phase cells indicating an S phase-specific wave of transcription through the upstream *ORG* cluster, consistent with a large continuous transcript into the *β*-globin locus. Combined with the EST data, these results suggest that large transcripts initiate from the LTR promoter early in S phase, immediately after the locus has replicated and process through the entire *ORG* cluster, LCR and into the *β*-globin locus. These transcripts are distinct from the bulk of transcription that occurs in the active LCR and δβ sub-domains that occurs predominantly in G1 phase. These results link high levels of non-S-phase intergenic transcription with chromatin domains that are highly enriched in active histone modifications.

## Discussion

We have shown that the human *β*-globin locus is composed of multiple chromatin sub-domains that are developmentally regulated. The individual sub-domains can be distinguished by differential general sensitivity to DNase I, intergenic transcription and active histone modifications primarily to H3. Our data show that H3K4 di- and tri-methylation and H3 hyperacetylation clearly mark domains with high levels of G1 phase intergenic transcription. H4 hyperacetylation also marks the active domains but appears at moderate levels in the inactive sub-domains in embryonic and adult erythroid cells in transgenic mice. We show that a very large transcript that initiates approximately 236 kb upstream of the human *β*-globin locus and extends through the locus is produced primarily in S phase. Transcription of the active sub-domains, containing the LCR and the active genes at each developmental stage, occurs primarily in G1 phase. These results correlate high levels of G1 phase-specific intergenic transcription with high levels of active histone modifications, namely, H3K4 di- and tri-methylation, and H3 acetylation, across the transcribed sub-domains, suggesting that the timing of intergenic transcription and/or the level of transcription may play a role in propagating these marks. There are clear indications that the elongating form of RNA polymerase II (RNAPII) is associated with histone modifying and chromatin remodelling activities[38], which could account for the modified domains we observe in areas of high intergenic transcription. Additional possibilities are suggested by recent studies indicating that transcription by RNAPII outside of S phase could promote replication independent histone exchange leading to the deposition of variant histones such as H3.3[39]. Although non-genic transcription is widespread in vertebrate genomes it is unlikely that all of this transcription leads to deposition of variant histones and active histone modifications. Controlling the timing of intergenic transcription may be a strategy adopted to modify specified domains.

In adult erythroid cells the LCR and the adult *β*-globin genes engage in long-range interactions, essentially forming a chromatin loop[40], [41]. The mechanism by which these distal sequences find each other has been the subject of intense speculation and debate. Although a loop is formed, a “looping mechanism” implies that the two sequences find each other via diffusion-mediated random collisions. The discovery of intergenic transcripts initiating in the LCR and proceeding in the direction of the globin genes have suggested a tracking mechanism of enhancer-gene contact in which the LCR and associated factors including RNAPII track through the locus in search of an activatable gene promoter. Our data indicate that the majority of LCR transcripts are not contiguous with δβ domain transcripts, implying that a continuous scanning mechanism is unlikely. However, a low level of transcription through the εγ domain may be contiguous with LCR and δβ domain transcripts. If the chromatin loop was established through a scanning mechanism one might expect that minimally it would need to operate at least two times per cell cycle. First, when cells exit mitosis, long-range contacts may need to be re-established after decondensation of the inactive mitotic chromosome structure prior to gene transcription. Second, after DNA replication, which is known to temporarily disrupt transcription, long-range contacts may need to be re-established on individual daughter alleles in early S-phase. Although we do not know the precise timing of G1-specific intergenic transcripts in the εγ domain (i.e. early or late G1) intergenic transcripts are clearly present there in early S-phase just after replication of the locus, suggesting that a limited tracking mechanism of LCR-gene interaction would be compatible with our data. However, a diffusion-mediated looping mechanism of LCR-gene contact is not ruled out by our results.

What could be the role of the very long transcripts that initiate at −236 kb upstream of the locus? The null hypothesis is that it has no role at all and is merely the result of transcriptional noise. However, the transcript appears to be very tightly regulated occurring predominantly in S phase, strand-specific and apparently initiating from a single discrete site. It is possible that histones in the *ORG* and εγ domain regions are modified by passage of the RNAPII complex as part of decondensation of the globin locus, but that active marks in these regions are rapidly or more thoroughly turned over due to the rarity and very low level at which these transcripts occur. We estimate that there may be as little as a single RNAPII complex transcribing these regions in loci with a positive RNA FISH signal. ChIP analysis on a sorted or synchronized population of early S-phase erythroid cells could address the question of whether this low level transcription is linked to transient histone modifications.

Another potential role of intergenic transcription is to facilitate the re-entry of the globin locus into a transcription factory after DNA replication. DNA replication takes place in replication factories which form in proximity to transcription factories in early S phase[42]. In the latter case active genes may need to disengage from RNAPII factories to shuttle to a nearby replication factory. Highly-expressed genes like globin, which are nearly always associated with transcription factories in expressing cells[43], [44], could be reeled back into a transcription factory after replication by the processive action of RNAPII localized in a factory[45].

In summary, our data strengthen the link between intergenic transcription and modification of histones over wide chromatin domains, and suggest that developmental regulation of expression the human *β*-globin genes occurs in part through epigenetic changes to chromatin structural domains.

## Materials and Methods

### Animals and Human primary cell culture

Experimental procedures were conducted in compliance with an animal protocol approved by the home office and local ethical review committee. Transgenic mice homozygous for a wild type 150 kb human *β*-globin locus YAC were previously described by Tanimoto *et al*.[27]. Adult mice were made anemic as previously described[46]. Human peripheral blood from healthy individuals was obtained from a local blood bank, prepared and cultured as described in Chakalova *et al*.[6], and harvested on day 2 post-hemoglobinization.

### RNA-FISH

RNA FISH was performed as previously described[16]. The following probes were used to visualise intergenic transcription signals (coordinates relative to the *ε* gene transcription start site at position +1 are given in brackets): 5′UBQLN3 (-243679 to -241290), 3′UBQLN3 (-236996 to -236060), 5′ORG14 (-235344 to -232998), 3′ORG14 (-219479 to -221543), 3′ORG8 (-131754 to -130106), 3′ORG7A (-113405 to -111608), 3′ORG7B (-100614 to -99051), 5′ORG6 (-94181 to -92378), 5′ORG3 (-57154 to -55376), 3′ORG1 (-27315 to -25831), HS5 (-22844 to -19501), HS3 (-16236 to -14330), 3′ε (3126 to 5919), 5′γ (10881 to 12866), 5′δ (32851 to 35239), 5′β (38504 to 42384). Digoxigenin-labeled single-stranded DNA probes were generated from the cloned sequences as described[47] and detected with FITC-labeled antibodies. Intergenic probes were used in combination with a probe to the *ΗΒΒ* gene intron 2; in double-label RNA FISH experiments, a cocktail of four dinitrophenol-labeled *ΗΒΒ* intron 2 30-mer oligonucleotides were used (described in [6]), and detected with Texas Red-labeled antibodies; for three-colour cell cycle analysis, a longer biotin-labeled single-stranded DNA probe (coordinates 43126 to 44043) was applied and detected with Alexa Fluor 350-labeled Streptavidin (Molecular Probes Invitrogen). In all cases, slides were examined on an Olympus BX41 epifluorescence microscope. To determine the percentage of loci associated with intergenic transcription signals, a minimum of 100 loci were counted for each data point. PCNA was visualised with a primary mouse monoclonal anti-PCNA antibody (Santa Cruz) followed by Texas Red-conjugated goat anti-mouse secondary antibody (Jackson ImmunoResearch). To determine the percentage of PCNA-positive intergenic transcription signal-containing cells, cells with intergenic signals were identified, and digital images were captured for each of the three colour channels to record gene and intergenic FISH signals as well as PCNA patterns. 40 cells were analysed for each data point.

### Quantitative RT-PCR

Total RNA was extracted from adult anemic spleen and day 11 embryonic blood of homozygous transgenic mice. RNA was isolated according to the manufacturer's instructions from frozen cell pellets using 4 ml of RNA-Bee (AMS Biotechnology) per 10^7^ cells. 1 µg of total RNA was mixed with Random hexanucleotide mix (5 ng/µl final concentration, Promega) and RNase-free water in a final volume of 20 µl. Reverse transcription was carried out with Superscript II reverse transcriptase (Invitrogen) following the protocol provided by the manufacturer in the presence of 2 u/µl RNasin. RT negative controls in which the reverse transcriptase enzyme omitted were set up in parallel. Real-time PCR was performed with an ABI PRISM 7000 Sequence Detection System using SYBR green PCR Master Mix (Applied Biosystems). 2 µl of cDNA were used in real-time PCR, in duplicate, with the following thermal cycling conditions: 50°C for 2 minutes and 95°C for 5 minutes, followed by 40 cycles of 95°C for 30 seconds and 62°C for 2 minutes. For primer sequences, see Table S1 in Supporting Information. The relative amount of cDNA amplification for each primer pair was calculated by comparing to transgenic genomic DNA standards. Data were normalised to the most 5′ data point in the olfactory receptor region, which shows low level of transcription.

### Chromatin immunoprecipitation

Histone modification profiles across the human *β*-globin locus were assessed by native chromatin immunoprecipitation (NChIP)[48]. Single-cell suspensions of erythroid or mouse embryo fibroblast cells were resuspended to 2×10^7^ cells/ml in ice cold 1×RSB (10 mM Tris-HCl, pH7.5, 10 mM NaCl, 3 mM MgCl_2_), 0.1% Triton X-100, 0.5 mM DTT, 0.1 M sucrose, 0.1 mM PMSF (Sigma), 5 mM Na butyrate and 1/50^th^ volume protease inhibitor cocktail (Sigma). The cells were dounced in a cold glass homogeniser and diluted with an equal volume of the same buffer with 0.25 M sucrose. The suspension was layered onto a sucrose cushion consisting of a half volume of 0.33 M sucrose, 5 mM MgCl_2_, 10 mM Tris pH8, 0.5 mM DTT, 0.1 mM PMSF, 5 mM Na butyrate. This was then centrifuged at 800 x g for 5 min at 4^o^C to obtain the nuclear pellet.

After preparing nuclei, chromatin was digested with micrococcal nuclease generating DNA predominantly mononucleosomal in length. NChIP was carried out using the following rabbit polyclonal antibodies: anti-trimethyl-histone H3 (K4) (Abcam), anti-dimethyl-histone H3 (K4), anti-acetyl-histone H3 (K9/K14), anti-acetyl-histone H4 (K5/18/12/16), ChIP-grade anti-hyperacetylated histone H4, penta lysine (all from Upstate Biotechnology)

DNA from the Input chromatin fractions was quantified by standard spectrophotometry. DNA concentrations in the antibody-bound fractions were determined by PicoGreen (Invitrogen) fluorescence quantification, using Input DNA for standards. Real-time PCR was performed in an ABI PRISM 7000 Sequence Detection System using SYBR green PCR Master Mix (Applied Biosystems). All PCR reactions were carried out in duplicate on 3 ng DNA at 50°C for 2 minutes and 95°C for 5 minutes, followed by 40 cycles of 95°C for 30 seconds and 62°C for 2 minutes. For primer sequences, see Table S1 in Supporting Information. The ratio of Bound to Input DNA was calculated using the comparative C_T_ method, Bound/Input = 2^(Input Ct – Bound Ct)^ [49]. Data were normalised to the most 5′ data point in the olfactory receptor region, which shows low enrichment for all antibodies. The promoter of the ubiquitously expressed mouse *Actb* (*β*-actin) gene was used as an internal positive control (Supporting Information Figure S1). The ChIP experiments were repeated several times with similar results and domain patterns. Shown are the results of a single representative ChIP experiment.

## Supporting Information

Figure S1 Histone modifications at the mouse Actb (beta actin) promoter. Histone modifications were assayed by ChIP in 264W transgenic mice at two developmental stages as a positive control for the ChIP procedure. The ChIP material is the same as in Figures 2 and 3 (Embryonic and Adult, respectively). Briefly, chromatin from erythroid cells was immunoprecipitated with antibodies specific for trimethylated lysine 4 of histone H3 (H3K4me3), dimethylated lysine 4 of histone H3 (H3K4me2), acetylated histone H3 (K9/14, H3ac), and acetylated histone H4 (two different antibodies: ChIP grade antibody, K5/18/12/16, H4acCh; penta lysine, H4acP). The fold-enrichment of antibody-bound sequences was analysed by real-time PCR (Bound/Input) using a primer pair in the mouse Actb promoter.
(0.19 MB TIF)

Table S1 Primer pairs used for real-time PCR. Primer pairs used to amplify sequences in the human HBB gene cluster and flanking regions; primer names reflect the position of the amplicon relative to the HBE gene transcription start site at position +1 or known genomic elements. Actb Pr is the mouse β-actin promoter region amplicon. For, forward primer; Rev, reverse primer.
(0.04 MB DOC)
