# Supplementary figures and images for: Intergenic Transcription, Cell-Cycle and the Developmentally Regulated Epigenetic Profile of the Human Beta-Globin Locus
Source: PLoS One. 2007 Jul 18;2(7):e630. doi: 10.1371/journal.pone.0000630 (PMC1910613; doi:10.1371/journal.pone.0000630)

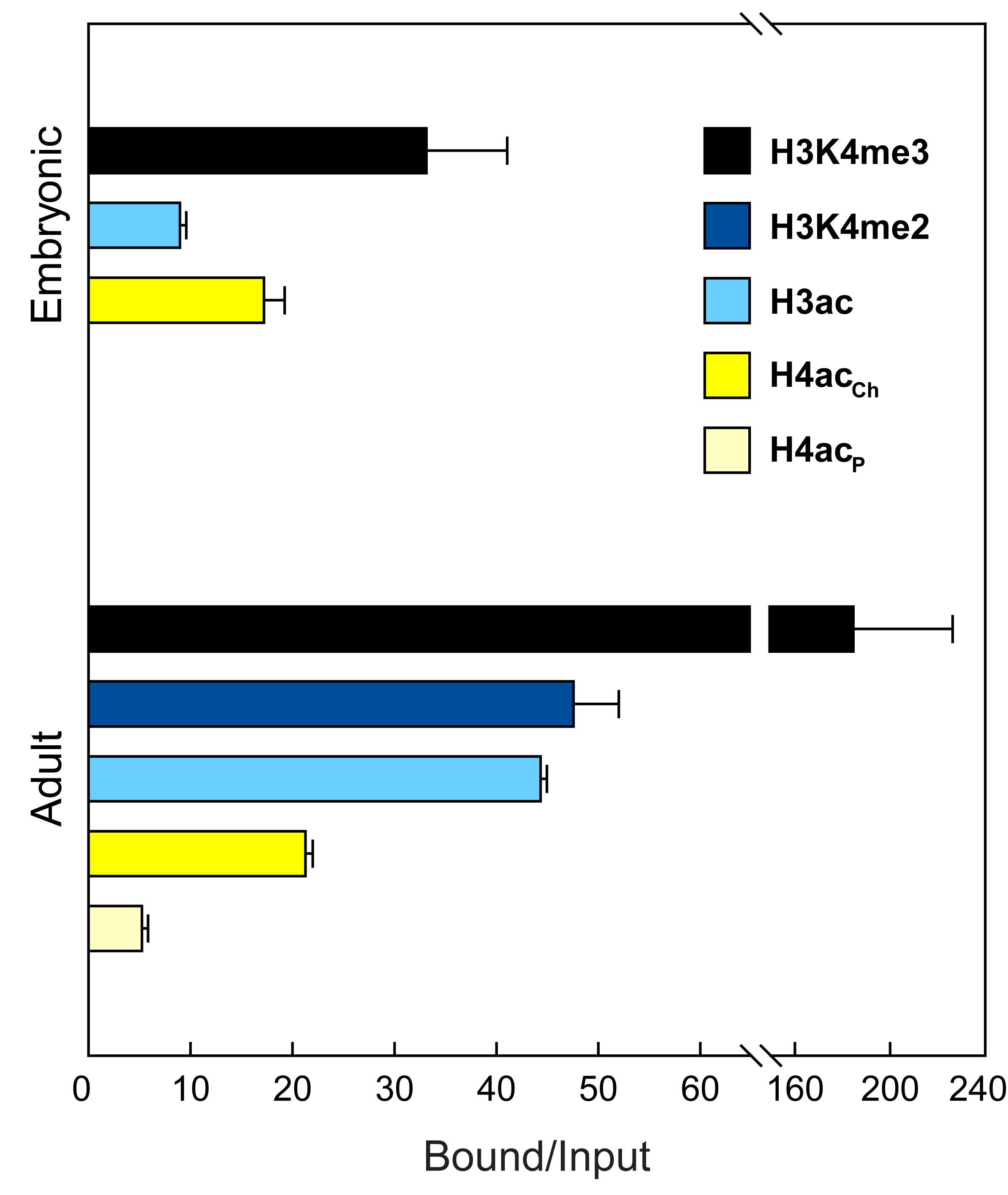

Supplement: Figure S1 — Histone modifications at the mouse Actb (beta actin) promoter. Histone modifications were assayed by ChIP in 264W transgenic mice at two developmental stages as a positive control for the ChIP procedure. The ChIP material is the same as in Figures 2 and 3 (Embryonic and Adult, respectively). Briefly, chromatin from erythroid cells was immunoprecipitated with antibodies specific for trimethylated lysine 4 of histone H3 (H3K4me3), dimethylated lysine 4 of histone H3 (H3K4me2), acetylated histone H3 (K9/14, H3ac), and acetylated histone H4 (two different antibodies: ChIP grade antibody, K5/18/12/16, H4acCh; penta lysine, H4acP). The fold-enrichment of antibody-bound sequences was analysed by real-time PCR (Bound/Input) using a primer pair in the mouse Actb promoter. (0.19 MB TIF) [file pone.0000630.s001.tif]
