# Supplementary material for: Intergenic Transcription, Cell-Cycle and the Developmentally Regulated Epigenetic Profile of the Human Beta-Globin Locus
Source: PLoS One. 2007 Jul 18;2(7):e630. doi: 10.1371/journal.pone.0000630 (PMC1910613; doi:10.1371/journal.pone.0000630)
Supplement: Table S1 — Primer pairs used for real-time PCR. Primer pairs used to amplify sequences in the human HBB gene cluster and flanking regions; primer names reflect the position of the amplicon relative to the HBE gene transcription start site at position +1 or known genomic elements. Actb Pr is the mouse β-actin promoter region amplicon. For, forward primer; Rev, reverse primer. (0.04 MB DOC) [file pone.0000630.s002.doc]

Primer Sequence Amplicon size, bp

p-237 For AGCTCCCTACCACTAGCCTGA 100

p-237 Rev ATCAAATCCTGGAAGTGCAAG

p-130.8 For TTACGCATCAACAGGGACCTC 102

p-130.8 Rev GGTCCTTGACATAGAGGTGTGCT

p-112.8 For ACCAGGACAGAGACCCATGAA 113

p-112.8 Rev ACACTCTGGCCCTAACAACCA

p-110.9 For GCTTAAATTCTGACCAGCACCA 93

p-110.9 Rev TATTGAGGACACGGGAAGGTG

p-110.3 For GTCCTTCCCAGAAGAACTCCA 111

p-110.3 Rev TGTGGACCAGTCCAGAAGACC

p-100.3 For CTCTGACCTGACAACCCAGCTA 103

p-100.3 Rev GCTGACTGTCCAGGATTGGAA

p-95.2 For GTTCTCACCACCAGCCCTTCT 96

p-95.2 Rev GAGTGTAGGGCTGGCAACTGT

p-56.4 For AGCTAAGCCATCTGAATGGAA 100

p-56.4 Rev TGAGGCCCACTATAAGCAGTG

p-52 For TCAAACCATGATAGAGCAGCA 100

p-52 Rev TTATCCAAAGTAAGCACCTCCT

p-45.8 For AGGATTGAGGCTGCAGAAATACA 110

p-45.8 Rev TTGTGTGTTCACAAGTGGCAAG

p-30 For ATTAGAAGGCCCATTTGGATG 70

p-30 Rev TCTCACAAACAATTCGCACAA

p-26.9 For CATCTGACAAATGTTTGAGGAACA 107

p-26.9 Rev GGAATTACAGATTTCGCCAGA

p-22.6 For TGTGAAGTGGTGGATTTGTTAT 74

p-22.6 Rev CAACATGATGCTTTGGTGTACG

p-22.3 For ATCATCACCATCCTGGACAA 74

p-22.3 Rev ATTTGAGCCAACATAGAGAGGAA

HS5 For CCAAGCAAGGAAGTTGTTAAGG 95

HS5 Rev AACTGGAGAAACTGGGAGGAG

p-20.6 For TTGAAAGGATTCTTACTTTGTGC 90

p-20.6 Rev ACCTGCCTTCTAGTGTTGAAAT

HS4 For AGCCATTGAAACAGGAATAACAG 112

HS4 Rev TCATGGACAATAACCCTCCTC

HS3 For GCAAGTGCCTTGACTCCTATG 88

HS3 Rev GTGCATCTCCTTTGATCCTCA

p-11.9 For CATGGCTGCCTAAAGTGTTGA 73

p-11.9 Rev TGCCAGTTAAGGTCTGCAGTG

HS2 For AGTGTTTAGCATCCAGCAGGT 85

HS2 Rev GAAGGGATAGAGGGAGCTGAG

p-10.1 For TGTAGGCAACTCTGATCCTGAGA 97

p-10.1 Rev TCATCCTCTTGTAGTCCTTCACA

HS1 For TGCGGTTGTGGAAGTTTAC 112

HS1 Rev TGTCACTAAGGGTGAGGATGC

p-3.4 For CTTGAACCAACAAGCATCGAA 113

p-3.4 Rev ATAGCATTGAAAGGCCCAGGT

p-1.9 For TTGTCTGTGTTAGCCAATGGT 95

p-1.9 Rev ATTCCAGGCCACTGAATTTCT

*HBE* Pr For AAGGAGAATGGGAGAGATGGA 97

*HBE* Pr Rev TGGCTCCTCATACTATCTGCAA

p2.1 For TAGCCTGCTCTTTCCATGAAG 90

p2.1 Rev TTGTATCAGAAGATAATGCACAA

p2.3 For GCATGAAACTGGCTCTCTGGA 69

p2.3 Rev TATCTGGTCTTTCCTAGTCCTCT

p12.2 For TGGCTTCTCACTCCCTACCAC 101

p12.2 Rev TGCCGTTGATATGTAAAGTGGA

p13 For ATGGAACCCAACCAGACTCTC 104

p13 Rev TAGAGACTGAGGTGAACCCAAA

p13.4 For CCTCAGTCTCTATAATCTGTACC 72

p13.4 Rev TGACTTCTCTCAATCTAAGGATG

PGFW For GAAGTCTGAAAGGATTCCACCA 74

PGFW Rev GGAGAGCTCTACCTTCCCCTAA

*HBG* Pr For CGGAACAAGGCAAAGGCTAT 102

*HBG* Pr Rev CAGGGACCGTTTCAGACAGAT

p17 For ACTATTTCCAACGGCATCTGG 86

p17 Rev CCTGTTTCCTGCTCTGATCTC

p17.3 For GTCAGCATCAGCGTGTCATGT 103

p17.3 Rev TCCAGTGAATCTGCCTTCTCA

*HBG* Pr For CGGAACAAGGCAAAGGCTAT 102

*HBG* Pr Rev CAGGGACCGTTTCAGACAGAT

p25.79 For CATTGTCACAACTCCTGATCCA 111

p25.79 Rev CAGCAGCCGCTAATAACCTGA

p25.8 For GGAATTGATGGCTAAGATATTCGT 84

p25.8 Rev GCCGCTAATAACCTGAATGCT

p26.4 For TCTGGGCAGGTAGGTACTGGA 109

p26.4 Rev CAGAGCAGGTGCAAAGTCAGA

p26.5 For ACTGTGCAATAATGGGCAACC 94

p26.5 Rev TTGAGGTCATCCGTGAGCATA

p26.7 For GGACCCTGAGAACTTCCTGGT 107

p26.7 Rev CAAGAGAAAGCCACCTCTGCT

p26.9 For GGAAGGCCACTTACCATTTGA 98

p26.9 Rev TCAACTTCTGTTCCCTCATCCA

p31 For TTAGTCAGCAAGCATGTGTCAT 107

p31 Rev TTGATGGACCCTAACTGATATAACTA

p31.2 For ACTAGGATGTGTCCAGTAAATGAAT 137

p31.2 Rev AATGCCAGTGCTCTCCACAAT

p31.7 For ACACCCAGCCAAGAATGTGAA 105

p31.7 Rev ATGTAAGGAGGATGAGCCACA

p31.8 For TTTCCCATCTTTCACCCTACC 97

p31.8 Rev GCTGTTGGTTTCAGAGCAGGT

p31.9 For GAACCTTATCTCCTACCTGCTCTGA 70

p31.9 Rev TGTAATGCCAATGTGGGTTAGAA

p32 For AACCTCTGACCTCCAACCTCA 110

p32 Rev CAAGTGAAATACCCATGCTGA

p32.2 For TCAGACTCAGCATGGGTATTTCA 98

p32.2 Rev CTTGGTGAATGGGCAAGTAGC

p33 For CCCAGCTATTTGTCTCCCATT 92

p33 Rev GGCATGGTTTGATTTGTGTCT

p33.3 For TGTGTTCACGACTGACATCACC 92

p33.3 Rev ATAGCTGGGCTTCTGTTGCAG

p33.4 For TGCAACAGAAGCCCAGCTATT 103

p33.4 Rev TGGCATGGTTTGATTTGTGTC

p33.5 For CGTTGGCTTCCAGGTTACAGA 119

p33.5 Rev TGGATGATGTGCCTGAGATTC

*HBD* Pr For ATGCAGAGGAGAACAGGGTTT 72

*HBD* Pr Rev TCCCTTAACTTGCCCTGAGAT

p38.4 For TTCCTACACCATTAGAAGTAGTC 115

p38.4 Rev TTGTGTTGCTCGGCACAT

5’*HBB* For TTCAAACTTCCGCAGAACACT 73

5’*HBB* Rev TCAAGACCCTGTTTCACATCC

*HBB* Pr For CACTTGCAAAGGAGGATGTTT 106

*HBB* Pr Rev TGGCTTAGGAGTTGGACTTCA

p45 For TAGCATTCAGGAAGAGATCAGAGG 69

p45 Rev GCAGAGCCAGAAGCACCATAA

p45.7 For GAATTGATGAACCTGGAGGATG 119

p45.7 Rev CTGTCTGTTTCCATGAGAGTGA

p64.1 For TGAGATGATTTATGTCATGGTAC 96

p64.1 Rev TGACATGGTCCATCTGTTCAA

p65.5 For TGGTGGCAACATGGATAATACTG 79

p65.5 Rev TTCATTCAGGCTTCTTCAGACT

*Actb* Pr For GTGACATCCACACCCAGAGG 108

*Actb* Pr Rev GAATAGCCTCCGCCCTTGT
